# The Proton Pump Inhibitor Lansoprazole Improves the Skeletal Phenotype in Dystrophin Deficient *mdx* Mice

**DOI:** 10.1371/journal.pone.0066617

**Published:** 2013-07-02

**Authors:** Arpana Sali, Gina M. Many, Heather Gordish-Dressman, Jack H. van der Meulen, Aditi Phadke, Christopher F. Spurney, Avital Cnaan, Eric P. Hoffman, Kanneboyina Nagaraju

**Affiliations:** 1 Center for Genetic Medicine Research, Children’s National Medical Center, Department of Integrative Systems Biology, George Washington University School of Medicine and Health Sciences, Washington, DC, United States of America; 2 Division of Cardiology, Children’s National Medical Center, Washington, DC, United States of America; Johns Hopkins University School of Medicine, United States of America

## Abstract

**Background:**

In Duchenne muscular dystrophy (DMD), loss of the membrane stabilizing protein dystrophin results in myofiber damage. Microinjury to dystrophic myofibers also causes secondary imbalances in sarcolemmic ion permeability and resting membrane potential, which modifies excitation-contraction coupling and increases proinflammatory/apoptotic signaling cascades. Although glucocorticoids remain the standard of care for the treatment of DMD, there is a need to investigate the efficacy of other pharmacological agents targeting the involvement of imbalances in ion flux on dystrophic pathology.

**Methodology/Principal Findings:**

We designed a preclinical trial to investigate the effects of lansoprazole (LANZO) administration, a proton pump inhibitor, on the dystrophic muscle phenotype in dystrophin deficient (*mdx*) mice. Eight to ten week-old female mice were assigned to one of four treatment groups (n = 12 per group): (1) vehicle control; (2) 5 mg/kg/day LANZO; (3) 5 mg/kg/day prednisolone; and (4) combined treatment of 5 mg/kg/day prednisolone (PRED) and 5 mg/kg/day LANZO. Treatment was administered orally 5 d/wk for 3 months. At the end of the study, behavioral (Digiscan) and functional outcomes (grip strength and Rotarod) were assessed prior to sacrifice. After sacrifice, body, tissue and organ masses, muscle histology, *in vitro* muscle force, and creatine kinase levels were measured. Mice in the combined treatment groups displayed significant reductions in the number of degenerating muscle fibers and number of inflammatory foci per muscle field relative to vehicle control. Additionally, mice in the combined treatment group displayed less of a decline in normalized forelimb and hindlimb grip strength and declines in *in vitro* EDL force after repeated eccentric contractions.

**Conclusions/Significance:**

Together our findings suggest that combined treatment of LANZO and prednisolone attenuates some components of dystrophic pathology in *mdx* mice. Our findings warrant future investigation of the clinical efficacy of LANZO and prednisolone combined treatment regimens in dystrophic pathology.

## Introduction

Dystrophin is located in the cytoplasmic portion of the sarcolemma and aids in muscle membrane stability and structural integrity. Dystrophin and other proteins of the dystrophin-associated complex serve as scaffolding proteins that regulate the association of proteins, such as ion channels, with the sarcolemma. Loss of dystrophin is thought to destabilize the myofiber membrane, in part, by altering the flux of ions and other proteins through the sarcolemma [1]. This is supported by observations of altered intracellular concentrations of Ca^2+^ [2], Mg^2+^ [2], K^+^ [3], and Na^+^ [4] within dystrophic myofibers. Such alterations in intracellular ion concentrations likely contribute to reductions in resting membrane potential, membrane resistivity, and excitability [5], [6] observed in in dystrophic myofibers, which negatively affects muscle function. Further, increases in intracellular Ca^2+^ levels within dystrophic fibers induce myofiber necrosis via increasing protease activity [7].

The progression of dystrophic pathology occurs as multiple mechanical and inflammatory insults contribute to chronic degeneration-regeneration remodeling cycles. Therapies aimed at reducing muscle inflammation, i.e. glucocorticoids, remain the standard-of-care for dystrophic pathology. Despite the acknowledged efficacy and widespread use of glucocorticoids, they do not fully ameliorate dystrophic pathology. Diets high in NaCl have been shown to attenuate myofiber necrosis and serum creatine kinase levels in *mdx* mice [8], suggesting that manipulation of systemic ion concentration gradients may help alleviate dystrophic pathology. This data also suggests a need to investigate the therapeutic efficacy of compounds capable of altering sarcolemmic ion flux.

Lansoprazole, 2-[(4-trifluoroethoxy-3-methyl-2-pyridyl)methylsulfinyl]-1H-benzimidazole, is a 369.36 kDa substituted benzimidazole that acts as a gastric proton pump inhibitor (PPI). Lansoprazole (LANZO) reduces stomach acidity by binding to hydrogen-potassium adenosine triphosphatase (H^+^/K^+^-ATPase) pumps located on the apical surface of gastric parietal cells. LANZO H^+^/K^+^-ATPase binding inhibits parietal cell secretion of hydrogen ions into the gastric cavity. LANZO is widely used in the treatment of gastroesophageal reflux disease (GERD) and peptic ulcers. In addition to reducing gastric acidity, LANZO has been observed to attenuate inflammation in the gastric mucosa [9], [10] and inhibit nitric oxide (NO) [11] production by macrophages, suggesting that an additional mechanism of LANZO action in the gut may include its anti-inflammatory properties. A similar PPI, omeprazole, has been shown to decrease the activity of immune cells in the peripheral blood after oral administration in healthy human subjects [11], suggesting LANZO may act systemically to attenuate inflammation. Inflammation is a major component of the dystrophic phenotypic in *mdx* mice and proinflammatory macrophage accumulation has been shown to lyse dystrophic fibers in an NO-dependent manner [12]. We thus sought to investigate the effects of this proton pump inhibitor on the skeletal muscle phenotype in *mdx* mice. We hypothesized that LANZO administration would attenuate the dystrophic skeletal muscle phenotype via altering ion influx and attenuating proinflammatory and apoptotic signaling cascades underlying the dystrophic pathology.

In this study, eight to ten week-old *mdx* mice were orally dosed with a saline vehicle control, LANZO, prednisolone, or a combined treatment regimen to determine the therapeutic efficacy of each treatment. After three months of treatment, dual LANZO and prednisolone administration was found to reduce serum creatine kinase levels, the number of degenerating muscle fibers, and the number of gastrocnemius inflammatory foci. Combined treatment of LANZO and prednisolone also improved *in vitro* and *in vivo* muscle force relative to vehicle control. Findings from our study, thus, warrant future investigation of the therapeutic efficacy of combined glucocorticoid and LANZO treatment regimens in the treatment of dystrophic pathology.

## Materials and Methods

### Animal Care

This protocol was approved by the Children’s National Medical Center Institutional Animal Care and Use Committee (IACUC) guidelines (Protocol # 1206) and thereby all animals were treated according to local IACUC standards. C57BL/10ScSn-Dmdmdx/J (*mdx*) 8–10 week-old female mice were purchased from Jackson Laboratories (Bar Harbor, ME). All mice were acclimated for 7 days prior to study initiation and individually housed in a vented cage system with 12 h alternating light-dark cycles. Mice received standard mouse chow and purified water *ad libitum*.

### Study Design

Four experimental treatment groups with 12 animals per group were included in this study: (a) vehicle control group receiving saline solution (0.9% NaCl, the vehicle used for LANZO administration); (b) experimental group treated with 5 mg/kg/day LANZO; (c) experimental group treated with 5 mg/kg/day prednisolone; and (d) combined treatment group receiving 5 mg/kg/day prednisolone and 5 mg/kg/day LANZO. Saline and LANZO were administered via oral gavage, and prednisolone was administered via oral drops. Each of the treatment regimens was administered 5 days per week (Monday-Friday) for 3 months. All functional, behavioral and histological assessments were acquired and analyzed in a blinded fashion.

### Treadmill Exercise

Treadmill exercise was performed on all mice before and during the 3-month treatment regimen to unmask the mild dystrophic phenotype of *mdx* mice based on previous studies demonstrating an exacerbation of the dystrophic phenotype in *mdx* mice with treadmill exercise [13]. All mice were exercised on a horizontal treadmill (Columbus Instruments, Columbus, OH) 2 days per week at a treadmill speed of 12 m/min for 30 min per day in accordance to the Treat NMD standard operating procedure for preclinical studies using *mdx* mice (http://www.treat-nmd.eu/downloads/file/sops/dmd/MDX/DMD_M.2.1.001.pdf). All exercise was performed between 0800 h and 1200 h and completed 1–2 days prior to functional testing.

### Rotarod Testing

Rotarod testing was performed according to methods we have described previously [14], [15], [16]. Briefly, mice were trained on a Rotarod apparatus (UgoBasile, VA, Italy) for 2 days prior to data collection on day 3. Each trial was performed twice per day, consecutively for 3 days, with a 2 h interval between sessions before and after the three-month pre-clinical trial. The latency to fall score was measured six times per trial for each mouse. The average latency to fall score was then calculated using data from each of the six trials and was expressed in seconds for each mouse tested.

### Grip Strength Testing

Forelimb and hindlimb grip strength was assessed at the beginning and end of the trial in order to evaluate changes in motor strength in response to the different treatment regimens. Forelimb and hindlimb grip strength measurements were performed using a Grip Strength Meter (Columbus Instruments, Columbus, OH). Grip strength was quantified by averaging five successful hindlimb and forelimb strength measurements each recorded within a 2 min time interval in order to calculate absolute grip strength. All strength measures were then normalized to individual animal body mass to calculate normalized grip strength, expressed in kgf/kg, as described previously [16].

### Behavioral Activity Measurement

Open field behavioral activity was measured using an open field Digiscan apparatus (Omnitech Electronics, Columbus, OH) in order to assess locomotor activity as described previously [16]. All mice were acclimated 60 min prior to data collection. Outcome variables included total vertical and horizontal activity, total distance traveled, movement time, and rest time. Data were collected every 10 min over a 1 h period before and after the pre-clinical trial.

### Histological Evaluations

At the end of the study, all mice were euthanized via carbon dioxide exposure, and organ and tissue samples were collected for histological evaluation. After weighing, a portion of each of the dissected tissues (i.e., gastrocnemius, EDL, soleus, heart and spleen) was kept in formalin for H&E staining. The remaining portion of each tissue was embedded in OCT compound and frozen in isopentane chilled in liquid nitrogen. Sections were imaged using a Nikon E800 microscope and taken at a magnification of 20x. Five non-overlapping field images representative of the overall tissue histology were captured using digital imaging computer software (Olympus C.A.S.T. Stereology System, Center Valley, PA). All of the digital images were uploaded into Image J Software (NIH, Bethesda, MD) for analysis using a cell counter software plug-in. The number of inflammatory foci and fibrosis were assessed in the diaphragm between treatment groups. Diaphragm fibrosis was assessed by use of Picro sirius red staining with a Weigert's haematoxylin nuclear counter stain on diaphragm cross-sections. In gastrocnemius cross-sections the total number of degenerating, regenerating and inflammatory foci per field were quantified in order to assess differences between treatment groups. The average number of fibers sampled in the 5 non-overlapping cross-sections was 170, with a range of 88–300 fibers per field. Fibers displaying a loss of striations/homogenous appearance of fiber contents were counted as degenerating fibers. Regenerating fibers were counted as fibers with basophilic cytoplasm, large peripheral, or central nuclei with prominent nucleoli. All cell counts per image field (degenerating/regenerating fibers and inflammatory foci) were assessed in a blinded manner as described previously [16].

### Creatine Kinase Determination

Blood for creatine kinase (CK) assay was collected by cardiac puncture into preservative-free Eppendorf tubes immediately after animal sacrifice. Blood serum was isolated by centrifugation for CK activity determination by standard enzymatic assay preformed according to the manufacturer’s instructions (CK10, Fisher Scientific). Enzymatic activity was calculated by measuring light absorption on a spectrometer set at 340 nm every minute for a total of 2 min at 37°C. All serum samples were run in duplicate for CK quantification.

### *In Vitro* Force Measurement

*In vitro* force measurements were performed according to methods we have described previously [17]. Briefly, the EDL of the right hindlimb was surgically removed and placed vertically in 25°C bath containing buffered mammalian Ringer solution and aerated with 95% O_2_–5% CO_2_. Muscles were secured to the lever arm of a servomotor/force transducer by the distal tendon (model 305B, Aurora Scientific) and the proximal tendon was connected to a stationary post in the bath. The muscles were stimulated at optimal length for 300 ms. Increasing electrode frequencies were used until a plateau was achieved and recorded as the maximal force. Specific force was obtained by dividing the maximal force by the EDL cross sectional area. Additionally, the muscles were subjected to a protocol of 4 lengthening contractions separated by 1 min intervals of rest. The muscles were lengthened over 10% of their length.

### Statistical Analysis

All analyses were performed using Stata V11 (College Station, TX). Results are represented as mean±SEM or median (range), where appropriate. Normality was assessed for each measurement using the Shapiro-Wilk normality test. For measurements meeting the assumption normality, analysis of variance (ANOVA) was used to compare means among groups (vehicle control, LANZO treatment, prednisolone treatment and combined treatment). *Post-hoc* pairwise group comparisons were performed where ANOVA models showed a significant F-test and the resulting group p-values were adjusted for multiple comparisons using the Sidak method. For measurements not meeting the assumption of normality (e.g. baseline open field measures), a non-parametric Kruskal-Wallis test was used to compare medians among groups. Data displaying significant p-values from the Kruskal-Wallis was further analyzed for between group differences via Wilcoxon rank-sum pair-wise comparisons. Resulting p-values were again adjusted for multiple comparisons using the Sidak method. Rotarod data was analyzed as a time to fall analysis using a log rank test of the equality of survivor functions between treatment groups. CK values were log-transformed to conform to normality. Histology measurements were compared between treatment groups using Poisson regression models for count data where appropriate. Longitudinal analysis of the repeated lengthening contractions used a mixed-effects linear regression model where mouse ID# was a random coefficient and where each treatment was compared to control only. Nominal statistical significance was set at α = 0.05.

## Results

### Effect on Muscle and Organ Weight

Mice in each of the treatment groups gained body mass over the course of this three-month pre-clinical trial (Table S1). Although mice were randomly assigned to treatment groups, there were statistically significant between-group differences in mean body mass prior to treatment (not shown). Due to these differences, baseline body mass was used as a covariate when comparing between group differences in mean body mass at the end of the three-month preclinical trial. Upon analysis, no significant differences in mean body mass between groups were observed at trial’s end (Tables 1 & S1). When comparing changes in mean body mass over the length of the trial, mice in the LANZO, prednisolone and combined treatment groups gained significantly less weight over the course of the study relative to the vehicle control (percent body mass gain relative to vehicle control: LANZO -23%, PRED −27%, Combined −36%) (Table S1). These findings support the efficacy of the treatment regimens as they are in agreement with our previous observations that untreated *mdx* mice gain more weight overtime than wild type littermate controls [14]. Muscle and organ weights were recorded at time of sacrifice. No significant differences in average gastrocnemius, soleus, EDL, or heart mass were observed between treatment groups (Table 1). A significantly lower mean spleen mass was observed in mice in the prednisolone and combined treatment groups relative to the vehicle control (spleen mass at time of sacrifice relative to vehicle control: PRED −22%, Combined −28%). Mice in the vehicle control and LANZO groups displayed similar spleen weights at time of sacrifice (94.3±4.2 and 95.0±6.0 mg, respectively) (Table 1). Additionally, the combined treatment group did not show a significant difference in mean spleen mass relative to the prednisolone treatment group (Table 1).

**Table 1:** Comparison of muscle and organ weights between treatment groups at time of sacrifice.

	Vehicle		LANZO		PRED		Combined			
Tissue Weight	N	Mean ± SEM	N	Mean ± SEM	N	Mean ± SEM	N	Mean ± SEM	Overallp-value	Significantly different means
Total Body Mass* (g)	10#	24.9±0.63	12	24.0±0.33	12	24.1±0.33	12	23.9±0.54	NS	None
Gastrocnemius (mg)¥	10	134.9±2.9	12	135.6±1.8	12	131.7±3.8	12	126.2±3.1	NS	None
Soleus (mg)¥	10	8.2±0.4	12	8.2±0.2	12	8.3±0.3	12	8.3±0.3	NS	None
EDL (mg)¥	10	10.3±0.4	12	11.4±0.4	12	11.7±0.4	12	11.1±0.2	NS	None
Heart (mg)	10	82.6±1.4	12	81.0±2.1	12	82.3±1.5	12	84.0±2.7	NS	None
Spleen (mg)	10	94.3±4.2	12	95.0±6.0	12	72.9±4.2^b, ab^	12	68.3±5.7^c, ac^	<0.001	^b^Veh vs. PRED (p = 0.038); ^c^Veh vs. Combined (p = 0.007); ^ab^LANZO vs. PRED (p = 0.021); ^ac^LANZO vs. Combined (p = 0.003)

Footnotes:

* Body mass was significantly different between groups at baseline, therefore, baseline body mass was included as a covariate in ANOVA modeling.

# Two of the mice in the vehicle control group died before the time of sacrifice and thus 10 mice were analyzed at study completion in the untreated group.

¥ The muscle weights presented represent average weight between right and left muscle groups.

### Effect of Treatment on Muscle Function

**Figure 1:**
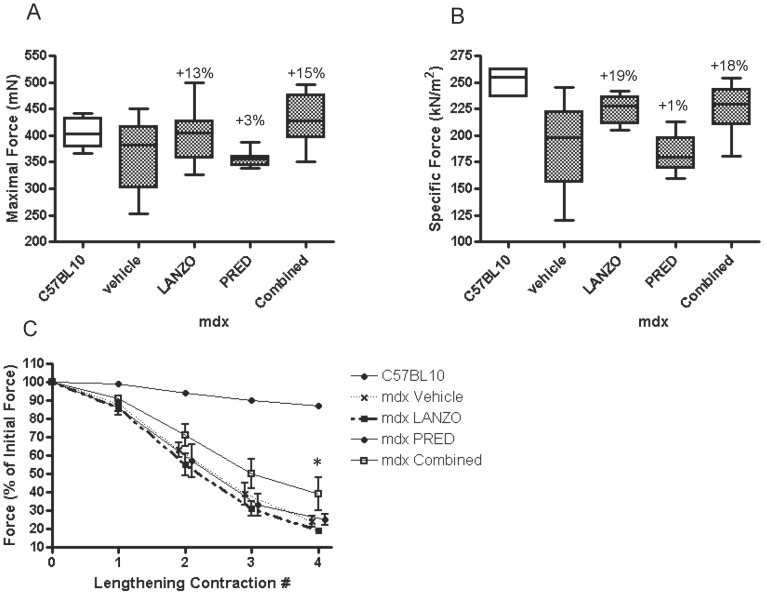
Effect of treatment on in vitro muscle force. The maximal (A) and specific (B) EDL muscle force was compared between vehicle control (n = 8), LANZO (n = 7), prednisolone (n = 8), and combined treatment groups (n = 9). The percent differences in maximal or specific force relative to vehicle control are presented above the error bars. No statistically significant differences in maximal muscle force were detected within treatment groups. There were statistically significant within group differences in specific muscle force (p = 0.0242), although no statistically significant between group differences were observed. (C) The EDL muscles of C57Bl/10 and *mdx* mice in each of the four treatment groups were subjected to four lengthening contractions and the decline in maximal force was tested using a mixed-effects linear regression model. Maximal force was also shown to decrease after each subsequent contraction due to the damage incurred by the lengthening contractions. *The overall decline in maximal force was significantly lower in response to repeated lengthening contractions in the combined treatment group relative to vehicle control.

*In vitro* maximal and specific muscle force was compared between treatment groups. Maximal force was not significantly different among treatment groups (p = 0.179), although there was a non-significant 13% and 15% increase in maximal force in LANZO and combined treatment groups, respectively, relative to the vehicle control group at the trial’s end (Fig. 1 A). Maximal force was not different between normal C57Bl/10 mice and vehicle treated *mdx* mice, which is consistent with previous reports [18]. However, there was a distinct reduction in EDL specific force in *mdx* mice in comparison to C57Bl/10 mice (Fig. 1 B), which is consistent with previous reports of reduced specific force in *mdx* mice [19]. *In vitro* specific force was significantly different among treatment groups (p<0.05). Although no statistically significant between group differences were observed, mice in the LANZO and combined treatment groups displayed a 19% and 18% increase in specific force, respectively, relative to the vehicle control at the trial’s end (Fig. 1 B). Isolated EDL muscle was additionally subjected to four lengthening contractions in order to assess declines in maximal muscle force in response to repeated eccentric stretching. Declines in maximal EDL force in response to lengthening contractions were significantly less in the combined treatment group relative to vehicle control (Fig. 1 C).

Forelimb and hindlimb grip strength testing was performed before and after the 3-month study and on untreated (non-exercised) C57Bl/10 mice, which served as a normal reference group. In *mdx* treatment groups, absolute forelimb grip strength was significantly different between groups prior to treatment (not shown). Therefore, baseline absolute forelimb grip strength was used as a covariate in ANCOVA models when determining between group differences in post-study grip strength measures. At the end of the pre-clinical trial, mice in the combined treatment group displayed a 10% higher absolute hindlimb grip strength relative to the vehicle control (p<0.01) (Table 2). Additionally, mice in the combined treatment group displayed a 12% higher absolute forelimb grip strength relative to mice in the LANZO treatment group (p<0.05). No other statistically significant between group differences in grip strength at the time of sacrifice were observed. There were no statistically significant differences in mean change in absolute forelimb or hindlimb grip strength (post minus pre values) between treatment groups (Table S1). Normalized grip strength did not display statistically significant differences between groups at trial’s end (Table 2). However, over the 3-month course of the study, mice in the prednisolone and combined treatment groups displayed an attenuated decline in normalized forelimb grip strength relative to the vehicle control (change in normalized forelimb grip strength relative to vehicle control: PRED +60% and combined +57%; p<0.05). Additionally, mice in the combined treatment group displayed a 44% attenuation in the decline of normalized hindlimb grip strength relative to the vehicle control at the trial’s end (p<0.05; Table S1).

**Table 2:** Effect of treatment on functional outcomes following 3 months of treatment.

	Vehicle		LANZO		PRED		Combined			
Measurement	N	Mean ± SEM	N	Mean ± SEM	N	Mean ± SEM	N	Mean ± SEM	Overallp-value	Significantly different means
Body weight (g)*	10	24.9±0.63	12	24.0±0.33	12	24.1±0.33	12	23.9±0.54	NS	None
Forelimb GSM (kgf)*	10	0.102±0.003	12	0.101±0.002	12	0.102±0.003	12	0.113±0.003^ac^	0.0368	^ac^LANZO vs. Combined (p = 0.049)
Hindlimb GSM (kgf)	10	0.185±0.004	12	0.198±0.004	12	0.192±0.003	12	0.204±0.003^c^	0.0099	^c^Veh vs. Combined (p = 0.009)
Normalized Forelimb GSM (kgf/kg)*	10	4.282±0.144	12	4.279±0.120	12	4.225±0.130	12	4.553±0.120	NS	None
Normalized Hindlimb GSM (kgf/kg)*	10	7.983±0.240	12	8.420±0.208	12	7.855±0.209	12	8.065±0.217	NS	None

Footnotes:

* Baseline measurements were significantly different between groups and thus baseline measurements were used as a covariate in ANOVA models.

### Effect of Treatment on Behavioral Outcomes

**Figure 2:**
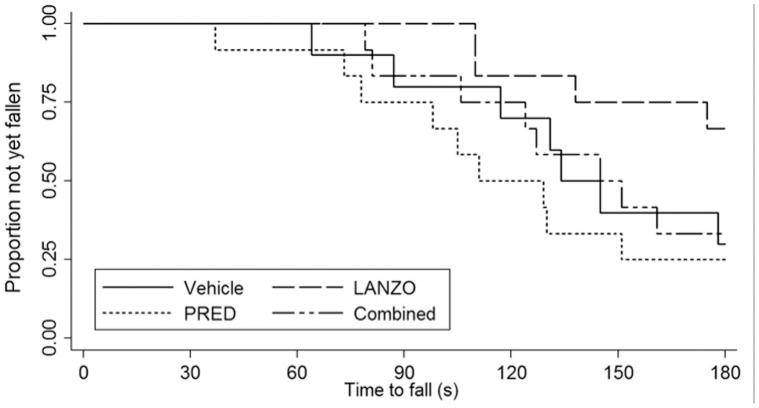
Effect of treatment on Rotarod latency to fall. Latency to fall time was measured between treatment groups (n = 12 per group, with the exception of the vehicle control [n = 10]) via Rotarod testing during the last week of the trial. The data analyzed is an average of six latency to fall scores where Rotarod testing was performed two times per day for 3 consecutive days after a 24 h acclimation period. Time to fall was compared between treatment groups using a log-rank test and Kaplan-Meier estimate. Although the LANZO and combined treatment groups displayed a greater median time to fall, no statistically significant differences were detected between treatment groups.

No statistically significant differences in post-treatment or changes in open field behavioral activity measures, including: total distance traveled, vertical activity, horizontal activity, movement time and rest time were detected between treatment groups (Table 3). Of note, there were no significant differences in baseline open field behavioral activity measurements (data not shown). There were no significant differences in median latency to fall time by Rotarod between groups at trial’s end by log-rank test (p = 0.115) (Fig. 2). However, mice in the LANZO treatment group did display a higher median latency to fall score relative to the vehicle control. Mice in the combined treatment group displayed similar latency to fall scores as mice in the vehicle control group, while mice in the prednisolone treatment group displayed the lowest latency to fall score.

**Table 3:** Effect of treatment on open field behavioral (locomotor) activity.

	Vehicle		LANZO		PRED		Combined		
Post-Treatment Measurement	N	Median (range)	N	Median (range)	N	Median (range)	N	Median (range)	Overall p-value
HACTV	10	1213 (785–2212)	12	1266 (772–2021)	12	1266 (995–1963)	12	1096 (402–1963)	NS
TOTDIST	10	251 (155–841)	12	273 (114–628)	12	253 (182–516)	12	213 (64–516)	NS
MOVTIME	10	27 (16–101)	12	29 (11–74)	12	28 (20–64)	12	25 (9–64)	NS
RESTIME	10	573 (499–584)	12	571 (526–589)	12	573 (536–580)	12	576 (536–591)	NS
VACTV	10	14 (10–88)	12	14 (7–37)	12	21 (12–47)	12	21 (3–47)	NS

Legend:

HACTV = total horizontal activity; TOTDIST = total movement distance; MOVTIME = total movement time; RESTIME = total rest time; VACTV = total vertical activity.

### Effect of Treatment on Muscle Histology and Creatine Kinase

**Figure 3:**
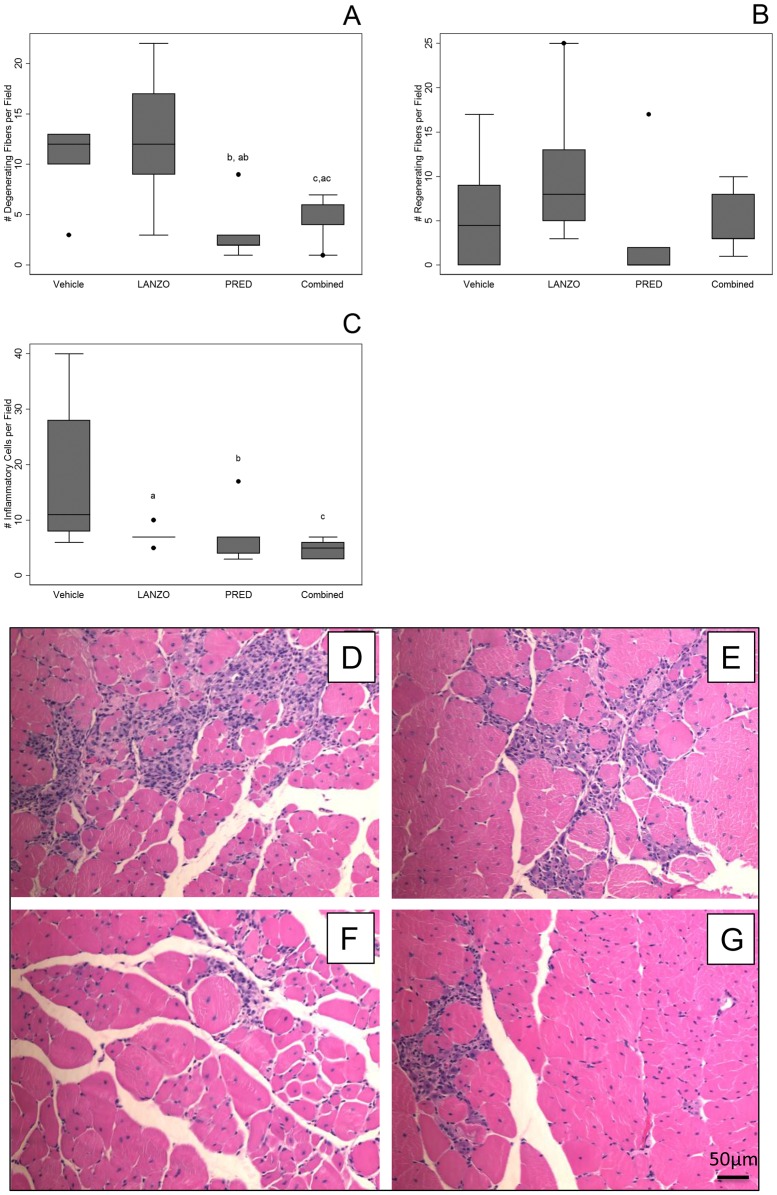
Effect of treatment on histological parameters between treatment groups at 20 weeks. H&E staining was performed on gastrocnemius muscle cross sections. Muscle histology (number of degenerating, regenerating and inflammatory foci per field) was averaged over five representative fields of non-overlapping fiber cross-sections (A–C). (A) Average number of degenerating fibers per field. (B) Average number of regenerating fibers per field. (C) Average number of inflammatory cell foci per field per group. (D–G) Representative gastrocnemius muscle cross sectional fields taken from vehicle control (D), LANZO-treated (E), prednisolone-treated (F), and combined treatment (G) groups.

Muscle histology was performed on gastrocnemius cross sections at time of sacrifice to quantify the number of degenerating/regenerating fibers and the number of inflammatory foci. Histology was compared between groups via Poisson regression on the median for count data, where appropriate. A decrease in the median number of degenerating fibers was observed in the prednisolone and combined treatment groups relative to mice receiving vehicle control (percent decrease in median number of degenerating fibers relative to vehicle: PRED −83%, p<0.01; combined: −50%, p<0.05) and LANZO treatment groups (Table 4; Fig. 3 A). No significant differences in the number of regenerating fibers were observed between any of the treatment groups (Table 4; Fig. 3 B). The median number of inflammatory foci per gastrocnemius cross sectional field was reduced in all three of the treatment groups relative to vehicle control (decrease in inflammatory foci per field relative to vehicle control: LANZO −36%, p<0.01; PRED −36%, p<0.05; combined −55% p<0.001) (Table 4; Fig. 3 C). Mice in the combined treatment group displayed the lowest amount of median inflammatory foci per gastrocnemius cross sectional field. Representative H&E stained gastrocnemius cross-sections are displayed in Fig. 3 D–G.

**Table 4:** Effect of treatment on histological outcomes following 3 months of treatment.

Measurement	Vehicle		LANZO		PRED		Combined		Significantly different means/medians	
	N	Median (Range)	N	Median (Range)	N	Median (Range)	N	Median (Range)		
Degenerating fibers per field (gastrocnemius)**	6	12 (3–13)	5	12 (3–33)	5	2 (1–9)^b, ab^	5	6 (1–7)^c, ac^	^b^Veh vs. PRED (p = 0.002); ^c^Veh vs. Combined (p = 0.018); ^ab^LANZO vs. PRED (p = 0.001); ^ac^LANZO vs. Combined (p = 0.005)	
Regenerating fibers per field (gastrocnemius) **	6	5 (0–17)	5	8 (3–25)	5	0 (0–17)	5	3 (1–10)	NONE	
Inflammatory foci per field (gastrocnemius) **	6	11 (6–40)	5	7 (5–10)^a^	5	7 (3–17)^b^	5	5 (3–7)^c^	^a^Veh vs. LANZO (p = 0.008); ^b^Veh vs. PRED (p = 0.012); ^c^Veh vs. Combined (p<0.001)	
Inflammatory foci per field (diaphragm)	6	59±16	6	56±16	6	57±17	6	46±15	NONE	
Fibrosis (diaphragm)	5	22.0±4.7	5	24.2±9.6	5	18.5±3.9	5	18.9±3.7	NONE	

Footnotes:

** Histology compared between groups using Poisson regression.

There were no significant between group differences in the mean number of inflammatory foci or fibrosis per diaphragm cross sectional field. However, the mean number of inflammatory foci was decreased ∼22% in the diaphragm of mice receiving combined treatment relative to vehicle control (Table 4). Additionally, a non-significant ∼14% decrease in the mean number of fibrotic regions was observed in the diaphragm of mice in the combined treatment group relative to vehicle control (Table 4).

**Figure 4:**
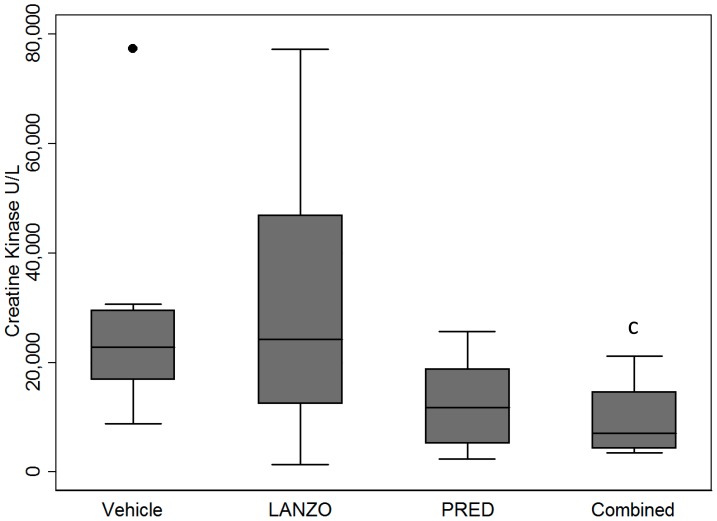
Effect of treatment on creatine kinase. Serum CK levels at time of sacrifice between vehicle control (n = 9), LANZO (n = 11), prednisolone (n = 12), and combined treatment (n = 11) groups. There were three mice for which a cardiac venipuncture was not obtained due to technical difficulties. All sera was obtained via cardiac venipuncture and analyzed in duplicate via enzymatic assay. Statistical analysis was performed on log-transformed data. Upon removal of two outliers in the vehicle and LANZO groups, no statistical differences remained among treatment groups (not shown).

Sera was obtained via cardiac venipuncture at time of sacrifice and analyzed in duplicate via enzymatic assay. Mice in the combined treatment group displayed a ∼64% reduction in mean serum CK relative to vehicle control (p<0.05) (Fig. 4). Mice in the prednisolone treatment group displayed a ∼54% reduction in mean serum CK relative to untreated control, but this did not reach statistical significance. No significant differences in mean serum CK were observed between any of the other treatment groups. However, upon removal of two outliers in the Vehicle and LANZO groups, no statistical differences remained among treatment groups.

## Discussion

Skeletal muscle excitability is regulated by ionic gradients. Following membrane depolarization, activation of the Na^+^/K^+^-ATPase proton pump restores resting Na^+^ and K^+^ gradients and helps maintain muscle excitability for repeated contractions [20]. Due to alterations in membrane stability and ion conductivity in dystrophic muscle, we sought to investigate the effects of the proton pump inhibitor LANZO on the dystrophic phenotype in *mdx* mice. The main finding of our study was that combined LANZO and prednisolone treatment improved some components of the dystrophic phenotype in dystrophin deficient *mdx* mice. Attenuation of the dystrophic phenotype in the combined treatment group relative to the vehicle control was indicated by observations of: 1) a decreased number of degenerating fibers and inflammatory foci per gastrocnemius cross sectional field; 2) attenuated declines in normalized forelimb and hindlimb grip strength; 3) attenuated declines in maximal *in vitro* EDL force in response to repeated lengthening contractions. LANZO administration decreased declines in body mass and reduced the number of muscle inflammatory foci. LANZO administration also appeared to improve specific and maximal gastrocnemius muscle force, although this did not reach statistical significance. The effects of LANZO and prednisolone were not additive, mice in the combined treatment group did display greater improvements in muscle force in response to repeated eccentric contractions and a reduced number of muscle inflammatory foci relative to the prednisolone treatment group.

The beneficial effects of dual LANZO and prednisolone administration are likely multifold. LANZO belongs to a class of proton pump inhibitors (PPIs) that become active in weakly acidic environments. LANZO has been suggested to bind to other classes of proton pumps, including the Na^+^/K^+^-ATPase [21]. However, the ability of the dose of LANZO used in this study to induce *in vivo* inhibition of proton pumps extragastrically has not been thoroughly investigated. We hypothesize that the mechanisms by which combined LANZO and prednisolone treatment improve the dystrophic phenotype in *mdx* mice may include: 1) reduced inflammatory cell recruitment to the skeletal muscle; 2) altered ion conductivity of skeletal muscle Na^+^/K^+^-ATPase and chloride ion channels; 3) decreased apoptotic signaling cascades promoting myonecrosis.

Off-label use of LANZO has been more thoroughly investigated in the treatment of cancer, wherein LANZO has been suggested to have anti-inflammatory and anti-metastatic effects. De Milito *et al.* observed that PPI administration promotes B cell apoptosis in acute B cell lymphatic leukemia via altering tumor lysosomes and extracellular pH [22]. The anti-metastatic effects of LANZO administration are also supported by a Phase I/II trial by Spungnini *et al.* who observed improved tumor outcomes in animals receiving combined treatment of LANZO and chemotherapeutics [23]. The ability of LANZO to inhibit tumor metastasis is suggested to occur via LANZO-mediated inhibition of V-ATPases, which are upregulated in metastatic cancers and contribute to maintenance of the metastatic tumor microenvironment and the Warburg effect via proton extrusion [23]. Both V- and H^+^/K^+^-ATPases are inhibited by disulfide binding to cysteine residues on their α subunits [24]. This supports the role of LANZO action on various classes of ATPases. Like the H^+^/K^+^-ATPase, the Na^+^/K^+^-ATPase is a class IIC P-type ATPase which is expressed in skeletal muscle. LANZO has also been observed to inhibit the Na^+^/K^+^-ATPase, albeit at a higher IC_50_ than it inhibits the H^+^/K^+^-ATPase [21]. The H^+^/K^+^-ATPase and Na^+^/K^+^-ATPase pumps are highly homologous and display species conservation in their catalytic α subunit cysteine residues (LANZO binding sites) [25]. In the skeletal muscle, the Na^+^/K^+^-ATPase pump resides at the sarcolemma and t-tubule system, where it functions to restore membrane potential following contraction via ATP-mediated Na^+^ extrusion and K^+^ import. It is conceivable that LANZO may improve components of the dystrophic phenotype by binding to the skeletal muscle Na^+^/K^+^-ATPase and altering ion influx. Dystrophic fibers display an accumulation of intracellular calcium, which promotes myonecrosis [7] and reduces skeletal muscle force [26] via altering excitation-relaxation coupling [27]. Dietary NaCl supplementation has been shown to attenuate myofiber necrosis and reduce serum CK concentrations in *mdx* mice [8]. As predicted by the Nernst equation, an increase in intracellular Na^+^ that may occur via LANZO-mediated Na^+^/K^+^ ATPase pump inhibition may reduce intracellular Ca^2+^ influx. A reduction in intracellular Ca^2+^ would thus reduce myofiber lysis and improve the dystrophic phenotype in *mdx* mice. Such alterations in Ca^2+^ ionic flux due to LANZO administration may also affect macrophage polarization and NO production as macrophage production of NO is enhanced in the presence of Ca^2+^ ionophores [28]. Contrary to this hypothesis of Na^+^ -mediated Ca^2+^ extrusion, an increase in intracellular Na^+^ levels may increase intracellular Ca^2+^ by decreasing the electrogenic potential of the Na^+^/Ca^2+^ exchanger. This is supported by findings from Allen *et al.* who observed that elevated intercellular Na^+^ promotes Ca^2+^ influx in myocytes [29]. Thus, the beneficial effects of combined LANZO and prednisolone administration may occur by mechanisms other than regulation of Na^+^ and Ca^2+^ influx and warrant further investigation. In dystrophic fibers, decreases in membrane resistivity increase membrane conductivity of a variety of ions including Cl^−^ [3]. Increases in Cl^−^ conductance alter skeletal muscle membrane excitability [30]. Increased Cl^−^ ion flux in fibroblasts surrounding myofibers of *mdx* mice has also been shown to worsen the dystrophic phenotype [31]. LANZO has been observed to inhibit gastric chloride ion channels [32]. Our findings of an attenuated decline in *in vitro* muscle force in response to repeated eccentric contractions in mice given combined LANZO and prednisolone treatment could thus be due to altered Cl^−^, Na^+^ or Ca^2+^ conductance and subsequent enhanced muscle excitability in response to LANZO administration. Declines in *in vitro* muscle force in response to eccentric contractions have been attributed to failure in E–C coupling and thus our findings support a potential role for LANZO in improving dystrophic muscle excitability [33]. Further research investigating the kinetics of oral LANZO administration on skeletal muscle ATPases will help elucidate the potential mechanisms of LANZO action.

Another putative beneficial effect of LANZO on the dystrophic phenotype may be due to its anti-inflammatory properties. Macrophage pre-treatment with LANZO decreases nitric oxide (NO) synthesis and cell viability in response to stimulation by LPS [34]. Further, pretreatment of monocytes with LANZO has been shown to decrease LPS-induced TNFα and IL-1β expression via decreasing IκB-α and ERK phosphorylation [35]. A similar PPI, omeprazole, has been shown to decrease neutrophil chemotaxis and ROS production [36]. Further, omeprazole has been shown to decrease peripheral blood neutrophil phagocytic activity and oxidation 4 hours after oral administration in healthy human subjects [11]. Since the mechanisms of actions between different classes of PPI are similar, we hypothesize that oral LANZO administration may attenuate the dystrophic phenotype in *mdx* mice by reducing neutrophil recruitment and respiratory burst and by skewing of muscle macrophages away from a proinflammatory M1 macrophage phenotype. This is supported as populations of iNOS expressing muscle macrophages have been suggested to contribute to muscle cell lysis in *mdx* mice and shifts in muscle macrophage activation from a proinflammatory to anti-inflammatory/regenerative phenotype improve dystrophic pathology [12]. Further, depletion of neutrophils has been shown to attenuate components of the dystrophic phenotype in *mdx* mice [37].

LANZO irreversibly binds to cysteine moieties and its serum half-life is ∼1.5 hours. Due to the rapid catabolism of LANZO by the gastric environment and metabolism by the liver, the effects of LANZO administration are likely less pronounced in the skeletal muscle relative to the gastric epithelia. Therefore, we hypothesize that dual LANZO and prednisolone administration was efficacious due to partial inhibition of Na^+^/K^+^-ATPase pumps and chloride channels. The beneficial effects of dual LANZO and prednisolone administration were not additive. Glucocorticoids, like prednisolone, upregulate Na^+^/K^+^-ATPases [38]. We thus believe that the combined treatment was more efficacious than prednisolone administration alone because of the ability of LANZO to reduce elevated skeletal muscle Na^+^/K^+^-ATPase activity, due to prednisolone treatment, and further decreasing neutrophil and macrophage cytoxcity. Additional investigation of the dose-dependent effects of combined LANZO and prednisolone treatment are thus warranted as higher doses of LANZO may be more efficacious in the treatment of dystrophic pathology.

### Conclusions

Our findings suggest that combined LANZO and prednisolone treatment provides slightly improved therapeutic efficacy in treating the dystrophic skeletal muscle phenotype in *mdx* mice relative to prednisolone treatment alone. Complications of DMD may include other symptoms that may be improved with LANZO administration, such as delayed gastric emptying, gastric inflammation, smooth muscle fibrosis and dilation [39], [40]. Due to the therapeutic benefits of LANZO on the dystrophic phenotype, future investigation of the clinical efficacy of dual LANZO and prednisolone administration in human trials may be warranted.

## Supporting Information

Table S1 Changes in body mass and functional outcomes before and after treatment. The mean change (final-baseline) in each parameter tested is presented for all treatment groups.
(TIFF)
